# Supplementary material for: Out-of-balance growth enables cost-free synthesis of the flagellum and other proteins in a single bacterium
Source: eLife. 2026 Oct 28;15:RP110747. doi: 10.7554/eLife.110747 (PMC13716390; doi:10.7554/eLife.110747)
Supplement: Supplementary file 2 [file elife-110747-supp1.docx]

**Supplementary File 1A. List of plasmids**

| **Plasmid** | **Source** | **Description** |
| --- | --- | --- |
| **pPro2-Venus** | (Sassi et al., 2022) | SC101ori-KanR-Pro2_mVenusNB |
| **pPro4-Venus** | (Sassi et al., 2022) | SC101ori-KanR-Pro4_mVenusNB |
| **pProB-Venus** | (Sassi et al., 2022) | SC101ori-KanR-ProB_mVenusNB |
| **pProC-Venus** | (Sassi et al., 2022) | SC101ori-KanR-ProC_mVenusNB |
| **pProD-Venus** | (Sassi et al., 2022) | SC101ori-KanR-ProD_mVenusNB |

**Supplementary File 1B. List of strains**

| **Strain name** | **Promoter**  **Label** | | **Source** | **Description** |
| --- | --- | --- | --- | --- |
| **E98KFD** | WT | (Mark Kim et al., 2020) | | MG1655 IntS::ZeoR-PRNA1-mCherry E98K galK::AmpR-fliFp-CFP flhDC-YFP |
| **E98KFD Pro2_T7** | P1 | (Mark Kim et al., 2020) | | MG1655 IntS::ZeoR-PRNA1-mCherry E98K galK::AmpR-fliFp-CFP flhDC-YFP flhDp::Pro4 |
| **E98KFD Pro4_T7** | P2 | (Mark Kim et al., 2020) | | MG1655 IntS::ZeoR-PRNA1-mCherry E98K galK::AmpR-fliFp-CFP flhDC-YFP flhDp::Pro5 |
| **E98KFD Pro5_T7** | P3 | (Mark Kim et al., 2020) | | MG1655 IntS::ZeoR-PRNA1-mCherry E98K galK::AmpR-fliFp-CFP flhDC-YFP flhDp::Pro2 |
| **E98KFC** | WT | (Mark Kim et al., 2020) | | MG1655 IntS::ZeoR-PRNA1-mCherry E98K galK::AmpR-fliFp-CFP attb::KanR-fliCp-mVenusNB |
| **MGPro2-Venus** | P1 | (Sassi et al., 2022) | | MG1655 E98K + pPro2-Venus |
| **MGPro4-Venus** | P2 | (Sassi et al., 2022) | | MG1655 E98K + pPro4-Venus |
| **MGProB-Venus** | P4 | (Sassi et al., 2022) | | MG1655 E98K + pProB-Venus |
| **MGProC-Venus** | P5 | (Sassi et al., 2022) | | MG1655 E98K + pProC-Venus |
| **MGProD-Venus** | P6 | (Sassi et al., 2022) | | MG1655 E98K + pProD-Venus |
| **MC4100*** | - | This work | | MC4100 E98K ΔIS5_flhD flhD corrected galK::AmpR-fliFp-CFP flhDC-YFP |

Newly created strains (MC4100*) are described in Supplementary File 1B and are available from the corresponding author upon request, without restrictions.

**Supplementary File 1C.  Estimated mVenus proteome fraction across promoter strains.**

| **Strain** | **Promoter name** | **Average**  **Elongation Rate (h⁻¹)** | **% reduction vs. WT** | **Estimation**  **Φ_U_ (% dry cell weight)** |
| --- | --- | --- | --- | --- |
| **MGPro2-Venus** | P1 | 0.5618 | 1.29% | 0.55% |
| **MGPro4-Venus** | P2 | 0.5575 | 2.04% | 0.87% |
| **MGProB-Venus** | P4 | 0.5438 | 4.44% | 1.91% |
| **MGProC-Venus** | P5 | 0.5232 | 8.05% | 3.46% |
| **MGProD-Venus** | P6 | 0.51621 | 9.30% | 3.99% |

‘% reduction’ is calculated relative to the WT (non-expressing) growth rate $\mu_{max}$ =$0.5691 h^{-1}$). $\Phi_{U}$ is estimated using the ribosome-allocation relationship (Scott et al., (Scott et al., 2010)), with $b = 2.33 \mu g \mathrm{DCW}$ per µg protein, the empirically determined value for GFP-family proteins (Bienick et al., 2014 (Bienick et al., 2014)).
