# Supplementary material for: Out-of-balance growth enables cost-free synthesis of the flagellum and other proteins in a single bacterium
Source: eLife. 2026 Oct 28;15:RP110747. doi: 10.7554/eLife.110747 (PMC13716390; doi:10.7554/eLife.110747)
Supplement: Peer review history

# Editor report: eLife Assessment

**DOI:** 10.7554/eLife.110747.4.sa0

**Authors:** Ariel Amir

**Affiliations:** Weizmann Institute of Science Israel

**Keywords:** Convincing, Important

This **important** study addresses a discrepancy between population-level growth laws and single-cell correlations. It shows, for flagellar and synthetic genes in *Escherichia coli*, that while gene expression of certain genes reduces population-average growth, expression levels positively correlate with growth at the single-cell level. The measurements are **convincing**, and the proposed mechanism—inheritance of growth factors such as ribosomes during asymmetric division—can explain this observation.

# Referee report: Reviewer #1 (Public review)

**DOI:** 10.7554/eLife.110747.4.sa1

Summary:

Garcia-Alcala, Kratz and Cluzel investigate to what extent our understanding of bacterial physiology in bulk experiments can be applied to single-cell observations. They find that intrinsic noise may be powerful enough to even inverse the trends found in the bulk. The authors hypothesize that asymmetric distribution of ribosomes to daughter cells during the cell division plays the dominant role in the intrinsic noise and is able to generate the observed phenomenon. They do not show it directly, but the data and its agreement with the model suffice to support this claim.

Strengths:

The experimental part is convincing: the positive correlation between the elongation rate and promoter activity of unnecessary protein is clear, as well as the negative correlation between the mean values while changing the promoter strength. This was demonstrated in both rich and poor media. The causality between the growth rate and the promoter activity was shown using the negative lag time of the cross-correlation function. A simple, reasonable model accounts well for the data. This paper demonstrates an interesting phenomenon and provides a plausible theory for it, advancing our understanding of bacterial physiology on the single-cell level.

Weaknesses:

(1) Mean-reversion timescales were assumed to be longer than the simulation time and much longer than the cell cycle time. It is not clear whether the results robust in case mean-reversion timescales become of the order of cell cycle or smaller. If not, is there an argument for such practically infinite reversion timescales?

(2) It is not easy to understand the simulation part unless one reads Ref. [14]. Is k(t) assumed to follow Eq. (1) from ref. [14]? Is this crucial that the ribosome noise appears only at the division? The ribosome noise strength \sigma_R=0.06 - is it lower or higher than the naively expected binomial division?

Also, more intuitive explanation of the Simpson paradox would help the reader.

(3) It would be useful for the reader to see the raw data and not only the filtered one to appreciate the measurement noise level.

(4) Negative lag time of the cross-correlation function is visible, but consider adding statistical test for it.

(5) Can you make similar cross-correlation plots using the model? Can you infer using it whether the data agrees better with the assumption that ribosomes noise appear only at division or continuous fluctuations during the cell cycle?

Comments on revised version:

The authors addressed the five comments listed above.

# Referee report: Reviewer #2 (Public review)

**DOI:** 10.7554/eLife.110747.4.sa2

Summary:

The manuscript by Garcia-Alcala et al. reports an interesting paradox: the cost of gene expression slows the population-average growth rate, whereas at the single-cell level, expression levels from these genes positively correlate with the growth rate. The effect is observed in the expression of flagellar genes and a gene under a synthetic promoter in *E. coli*. The findings are explained by the inheritance of growth factors, including ribosomes, during asymmetric division.

Strengths:

(1) The manuscript adds strength to an emerging body of literature showing that the population-level bacterial growth laws do not match correlations based on single-cell data. The evidence presented here is more striking than in previous works (such as Pavlou et al., Nat. Commun. 2025), as the trends in population-level data and single-cell data are reversed.

(2) A relatively simple model correctly explains the trends in the data.

The findings raise an interesting question of whether similar effects occur in other bacterial species and, more broadly, in other organisms.

Comment on latest version:

The additional analysis has strengthened the conclusions.

# Author comment: Author response

**DOI:** 10.7554/eLife.110747.4.sa3

**Authors:** Mayra Patricia Garcia Alcala, Josiah C Kratz, Philippe Cluzel

**Affiliations:** Harvard University Cambridge United States; Carnegie Mellon University Pittsburgh United States; Harvard University Cambridge United States

The following is the authors’ response to the previous reviews.

**Public Reviews:**

**Reviewer #2 (Public review):**

(1) The differing behavior of the MG1655 and MC4100 strains remains a lingering question concerning the generality of the conclusions. It appears unlikely that ribosomes or other growth factors partition significantly differently in the MC4100 strain than in the MG1655 strain. Furthermore, based on Fig. S15, it is still unclear to what extent MC4100 exhibits growth-rate fluctuations, as stated in the text, rather than primarily size fluctuations, as shown in the figure. It is also unclear why such very slow fluctuations would lead to qualitatively different behavior, given that the proposed mechanism appears to be rather fundamental. It would be helpful for the authors to discuss these two points.

We thank the reviewer for revisiting this point. Here, we re-examined the MC4100 data in more detail and found that our original analysis was inaccurate; we now clarify this below along with new supporting analyses.

We agree that there is no a priori reason for ribosome or growth-factor partitioning to differ between MC4100 and MG1655, and our expanded analysis now supports this directly in Fig.S15.

Using the Class-1 promoter of the flagellar cascade in MC4100, which behaves under our conditions as a quasi-constitutive promoter, we find a positive correlation between instantaneous elongation rate and promoter activity that is comparable in magnitude to MG1655 (Fig.15C). We further analyzed publicly available data from two independent studies of strains expressing a constitutive fluorescent protein (new Fig.S18): Tanouchi et al. [44, 48], who tracked MC4100 at 25°C and 37°C, and Wang et al. [33], who tracked MG1655 and B/r strains. In these datasets, we found the same positive coupling between instantaneous elongation rate and expression, consistent with what we observe in MG1655. Together, these results indicate that the short-timescale coupling between growth-factor availability and gene expression that underlies our proposed mechanism is not strain-specific and is present in MC4100 for constitutively expressed and Class-1 activity.

On the nature of MC4100's slow fluctuations: We thank the reviewer for pointing out that Fig. S15 did not distinguish growth-rate from size fluctuations. We have now computed the autocorrelation function of elongation rate for both strains and added it to Fig. S15. This analysis shows that MC4100's characteristic oscillation is present in cell size but is absent from the elongation-rate autocorrelation. We have revised the text accordingly to state that MC4100 displays long-timescale oscillations in cell size, rather than describing this as a growth-rate phenomenon.

On why MC4100 does not show coupling between elongation rate and flagellar Class-2 activity despite this coupling being present at Class-1: In MC4100, the positive correlation between elongation rate and promoter activity, comparable to MG1655 at Class-1, is markedly attenuated at Class-2. Since both promoters are measured from the same cells and time series, this rules out our first assumption, a generic masking effect, and instead localizes the discrepancy to the Class-1-to-Class-2 step of the flagellar cascade, the pulse-generating switch we and others have described previously (Sassi et al., 2020, [24]). We do not have data to determine the molecular basis of this attenuation and present it as an open question.

(2) It is unclear what fraction of the total proteome mVenus represents in different measurements. Adding this information would strengthen the conclusions.

We agree that a direct proteomic quantification (e.g., Coomassie staining or mass spectrometry) would be the most direct way to determine the fraction of the proteome occupied by mVenus. However, we are no longer in a position to carry out such experiments.

As in our original response, our elongation-rate reductions (1.3-9.3% across our promoter series; see Table S3) remain far smaller than the 67% reduction associated with ~27% proteome occupancy reported by Scott et al. [6], making it unlikely that mVenus approaches such an extreme fraction in our system.

Now we used a validated quantitative model relating relative growth-rate reduction to unnecessary-protein fraction, built on the same ribosome-allocation theory as Scott et al. and empirically calibrated specifically for a GFP-family fluorescent protein (Bienick et al., 2014, PLoS ONE [59]). This model predicts μ/μ_max_ = 1 - b·ɸ_U_, with b = 2.33 µg dry cell weight (DCW) per µg protein determined for eGFP across multiple growth media. Applying this relationship to our own elongation-rate data, using the growth rate of our non-expressing WT strain as μ_max_, we estimate that mVenus constitutes approximately 0.6-4.0% of dry cell weight across our promoter series, reaching ~4.0% in our strongest-expressing strain, P5.

While this remains a model-based estimate rather than a direct proteomic measurement and involves some extrapolation across growth media and strain background relative to the original Bienick et al. study, both independent approaches converge on the same conclusion: mVenus occupies a modest fraction of the proteome in our experiments, well below the f_U_ = 14.4% upper value explored in our simulations. This supports our original framing of f_U_ = 14.4% as a conservative exploratory upper bound rather than an underestimate of experimental burden, and we have added this quantitative estimate (Table S3) to the revised manuscript to strengthen this point, as suggested.
